# Intestinal subocclusion due to colonic lipoma: a case report

**DOI:** 10.4314/pamj.v10i0.72232

**Published:** 2011-10-14

**Authors:** Kamal Bentama, Miloud Chourak, Ilias Chemlal, Mohamed Benabbou, Mohamed Raiss, Abdelmalek Hrora, Farid Sabbah, Abdesslam Benamer, Mohamed Ahellat

**Affiliations:** 1 Surgical Clinic C, University Hospital Ibn Sina, Rabat, Morocco

**Keywords:** Colon, endoscopy, occlusion, lipoma, colonoscopy, colectomy, benign tumor

## Abstract

Colonic lipomas are rare benign tumors infrequently met in clinical practice. Most of them are asymptomatic making frequent their fortuitous discovery. The therapeutic approach to the fortuitous discovery of a lipoma is even less clear. The treatment depends essentially on the clinical picture, on the size of the lipoma and on its location. We report the case of a 31-year old woman, which sub-occlusive accidents events revealed a lipoma of the descending colon. The diagnosis was suspected on colonoscopy and segmental colectomy was performed. The diagnosis was confirmed by histological examination. We review the literature and discuss the clinical features, diagnosis and treatment of this uncommon disease.

## Background

Colonic lipoma is a rare benign tumor. Colic lipoma was described for the first time by Bauer in 1757. The incidence of this lesion is estimated between 0.2 and 4.4% [1,2] and represents 1.8% of the colic benign lesions [3]. Approximately 320 cases are reported in the literature, mostly as case reports. We report a case of symptomatic lipoma of the descending colon in a 31-year-old woman. From this case and after review of the literature, we discuss the clinical characteristic, diagnostic and treatment options of this rare disease.

## Patient and case report

Mrs. X is 31 years old, without surgical history. The patient had presented within the past six months with violent episodes of spasmodic abdominal pain, aggravated by the recent installation of sub-occlusive episodes witch resolved spontaneously.

Clinical examination, including digital rectal examination at the time of the crisis revealed a diffuse distention with thickening at the left flank. Radiography of the abdomen without preparation and abdominal echography have not been of great contribution to orient to the diagnostic. Colonoscopy revealed a lesion in the left colon, six inches in diameter, obstructing the colonic lumen and covered by a whitish coating. This lesion was still passable and the colon upstream was free of any lesion.

Biopsies performed showed only a-cellular material largely necrotic. Abdomino-pelvic scan confirmed the existence of an intra-colonic mass of 55 mm in diameter, consisting mostly of fat tissue. No other abnormality was visualized on CT scan. There was no biological inflammatory syndrome, or anemia and tumor markers CEA and CA19-9 were normal. The diagnosis of colonic lipoma was suspected. With the persistence of sub-occlusive symptoms, we decided to operate.

**Figure 1:**
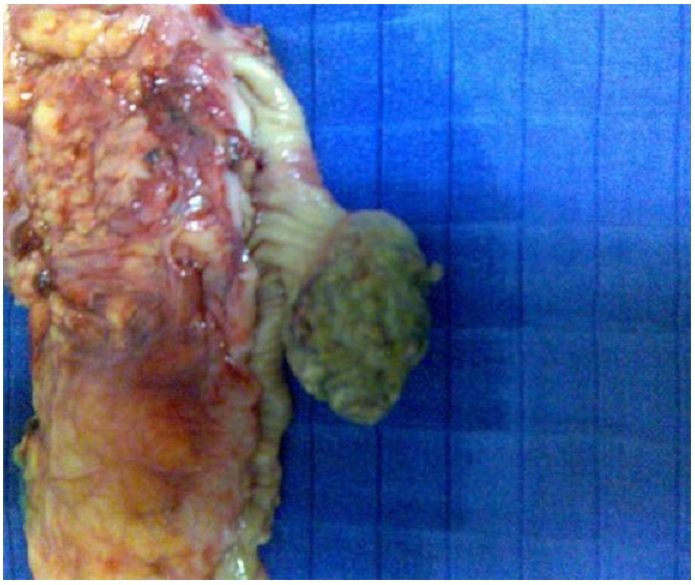
Surgical piece from patient operated for colonic lipoma showing a submucosal, pedunculated, fatty lipoma

**Figure 2:**
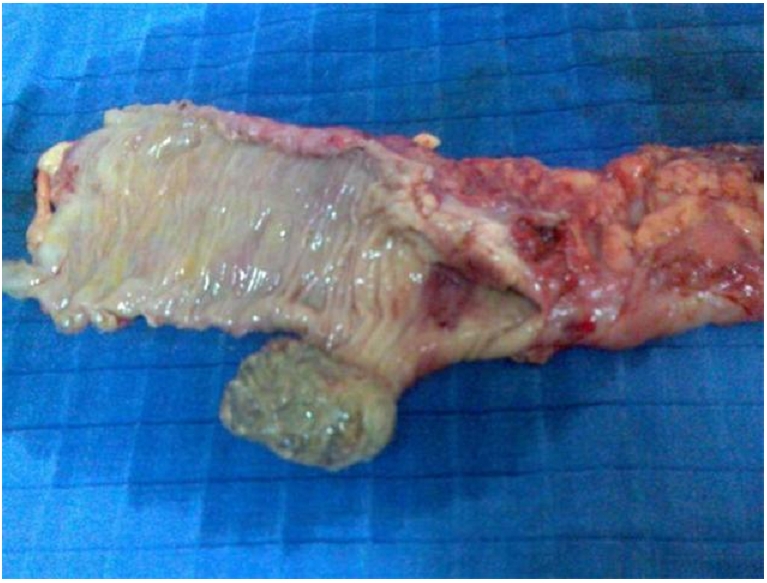
Surgical piece from a patient operated for colinic lipoma showing a lipomatous submucosal lesion lifting the colonic mucosa which is atrophic and ulcerated

The patient was operated by median laparotomy. Exploration discovered just below the splenic flexure a large mass, supple and mobile. Besides, there was no adenopathy or peritoneal nodules of carcinosis. A segmental colectomy was realized and allowed identification of the lesion which was fatty, submucosal and pedunculated (Figure 1 and Figure 2). Pathology confirmed the existence of a lipomatous proliferation lying below the colonic mucosa, which was atrophic and ulcerated and replaced by a fibrin-leukocyte rolling. The surgical outcomes were simple.

## Discussion

Colonic lipoma is the second most common benign tumor of the colon after adenomas. There is a feminine predominance, and the age of discovery is generally between 50 and 65 years [3,4]. The preferential localization of colonic lipoma is the right colon (61% of the reported cases), 15.4% on the transverse colon, 20.1% on the left colon, and 3.4% on the rectum [5]. In our case report, the patient was younger than that reported in the literature and the lesion was below the left colic angle.

The symptomatology is not specific. Only 6% of lipomas are symptomatic [5–7]. Most of the time, the discovery is fortuitous during a colonoscopy or on a colectomy [5,6]. The symptomatology is directly correlated to the size of the lipoma, often nonspecific, essentially consisting of abdominal pains, constipation and or rectal bleeding [5–9]. In a study conducted over a period of 34 years (from 1964 to 1998) that included the main series of the literature, approximately 282 cases showed abdominal pain to be present in 60% of the cases; transit disorders in 39% of the cases; and rectal bleeding in 31% of the cases. The lesions are the results of a purely mechanical phenomenon, the partial obstruction of the colon associated with occasional phenomena of colic intussusception are at the origin of mucous ulcerations [8–10].

One of the remaining challenges in the management of colonic lipoma is to establish the diagnosis preoperatively. Three diagnostic tools can bring arguments in favor of the diagnosis: colonoscopy allows mostly visualizing the lipomatous lesion characterized by a rise of the mucous membrane, softness of the mass under the biopsy forceps and finally the visualization of yellow fat on biopsy [10]. Barium enema can bring arguments in favor of the diagnosis, but its interest remains debatable considering the progress brought by CT scan. Indeed, abdominal scanner is currently the most reliable tool for the diagnosis of lipoma; it shows a regular, well bounded mass of greasy density [10]. Nevertheless, some atypical tomodensitometry images were reported making it difficult to differentiate it from a malignant tumor. In our case, the diagnosis was suspected in preoperative with colonoscopy, and confirmed by the histopathologic study of the operative specimen.

In front of a symptomatic lipoma, the treatment is the rule. Two options are possible: endoscopic excision or surgical excision. Certain authors suggest that the size of the lipoma is the limiting factor for endoscopic excision; the maximum size limit for endoscopic excision is fixed at most to 2.5 cm [7,10]; beyond that limit, the risks of bleeding and perforation seem too important. The surgical treatment remains the treatment of choice for big symptomatic and or complicated lipomas [10]. The surgical procedure used is dictated by the diagnostic certainty established in pre-op. Colotomy with lipectomy is the treatment of choice in the absence of complications [1,7]. In other cases, diagnostic doubt or acute intussusception, a segmental colic resection must be envisaged [1,7]. The therapeutic attitude in front of the fortuitous discovery of a lipoma is even less evident. Some people recommend the principal excision of the damage accessible to the endoscopy [7]. In the absence of diagnostic doubt, the most reasonable attitude in front of an asymptomatic lipoma is therapeutic abstention. Our patient had repeated subocclusive accidents in of the colon; we opted for a segmental colectomy. On histopathology, the lipoma develops in 90 % of the cases at the cost of the adipocytes under mucous membrane [5–7]; such was the case in our patient. More rarely, it develops under serous membrane. The lesion is mostly isolated, but multiple damage were reported in about 10% of the cases [6,7]. The prognosis of these lipomas of the colon is very favorable, no case of degeneration, no recurrence in case of complete excision, were reported [1,2,10].

## Conclusion

The lipoma of the colon is a benign and rare tumor. Most lipomas are asymptomatic, thus their frequent fortuitous discovery. The diagnosis is easy by colonoscopy coupled with biopsies. The treatment depends essentially on the clinical picture, on the size and location of the lipoma and involved endoscopic or surgical excision.
